# Efficacy of an intervention to increase therapeutic adherence in patients with secondary prevention for cardiovascular disease: a study protocol for a randomized controlled trial

**DOI:** 10.3389/fmed.2024.1510744

**Published:** 2025-01-07

**Authors:** Francisco Manuel Lidón-Muñoz, José Antonio Quesada, Vicente Francisco Gil-Guillén, Domingo Orozco-Beltrán

**Affiliations:** ^1^Dr. Balmis General University Hospital of Alicante, Babel Health Centre, Alicante, Spain; ^2^Department of Clinical Medicine, Miguel Hernández University of Elche, San Juan de Alicante, Spain; ^3^Research Network on Chronicity Primary Care and Prevention and Health Promotion, Alicante, Spain; ^4^Primary Care Research Center, Miguel Hernandez University of Elche, San Juan de Alicante, Spain

**Keywords:** cardiovascular diseases, chronic disease, secondary prevention, coronary disease, treatment adherence and compliance, primary health care

## Abstract

**Introduction:**

Cardiovascular diseases represent the leading cause of mortality worldwide. This category includes cerebrovascular disease, ischemic heart disease, and peripheral vascular disease. Secondary prevention is effective for patients with a history of cardiovascular events, with antihypertensives, statins, and acetylsalicylic acid being the most prescribed drugs. Therapeutic adherence is crucial, as the lack of it increases morbidity, mortality, reduces quality of life, and raises healthcare costs. However, less than half of patients adhere to all three drugs within the first year post-event. Furthermore, non-adherence is more pronounced in primary care.

**Objective:**

Based on our hypothesis that in patients undergoing cardiovascular secondary prevention, an intervention grounded in the application of the Chronic Care Model improves adherence to the three preventive drugs, this study aims to evaluate the efficacy of a health education intervention, which implements this model, to increase adherence in patients with cardiovascular disease.

**Methods:**

An open-label randomized controlled trial will be conducted, selecting patients who meet inclusion criteria through consecutive non-probability sampling. Random assignment will be performed using the random number table method. Based on a therapeutic adherence rate of 50% in the control group and 80% in the intervention group, with a type I error of 5% (95% confidence interval), a type II error of 20% (80% power), and accounting for a 15% loss to follow-up, the final sample size will be of 45 patients per group. Follow-up will last for 1 year. Following data collection, univariate, bivariate, and multivariate analyses will be performed to isolate confounding factors and assess the intervention’s impact on adherence.

**Discussion:**

If the results obtained in this study are favorable and the intervention is successful in enhancing therapeutic adherence, its applicability would be substantial, representing a feasible intervention for implementation in primary care. This approach addresses a significant public health issue, namely the lack of therapeutic adherence and its associated consequences. Moreover, this is particularly pertinent for high-risk patients, such as those in secondary prevention, given that cardiovascular disease remains the leading cause of mortality in developed countries. This trial has been registered at clinicalTrials.gov, https://clinicaltrials.gov/study/NCT06510946.

## 1 Introduction

Therapeutic adherence (TA) refers to the extent to which patients comply with therapeutic recommendations. The physician-patient relationship, once grounded in a paternalistic model, has evolved over the past few decades into a model of shared responsibility. Consequently, when discussing TA, co-responsibility serves as a fundamental pillar, acting as a cornerstone for decision-making and self-management of the disease. For this shift to occur, the relationship between patients and healthcare professionals must be based on trust and dialog.

Moreover, TA not only refers to the extent to which patients appropriately use drugs, but its dimension is also generally related to health. For this reason, it is necessary to distinguish between compliance, persistence, and TA (1), as these terms are not synonymous. Currently, it is preferable to specify the term lack of TA rather than noncompliance, since the latter implies that only patients are responsible for the treatment and the degree to which they appropriately follow the prescribed dosage. However, TA requires active patients who are knowledgeable about their disease, understand the prescribed treatments, and recognize the importance of adherence. Finally, therapeutic persistence relates to the time during which the patient continues the treatment, that is, the time that elapses from the initiation to the interruption of treatment (2).

The World Health Organization (WHO) defines adherence to long-term treatments by merging the concepts from Haynes and Rand as follows: “as the extent to which a person’s behavior–taking medication, following a diet, and/or executing lifestyle changes–corresponds to recommendations agreed with a healthcare provider” (3).

Cardiovascular disease (CVD) is the leading cause of early mortality across most European populations, including Spain (4), and is also the most common cause of mortality worldwide (5). Within the spectrum of CVD, cerebrovascular disease, ischemic heart disease, and peripheral vascular disease are included.

Secondary prevention is an effective strategy for patients who have experienced previous events, with the most commonly used drugs being antihypertensives, lipid-lowering agents, and antiplatelet and/or anticoagulant treatments, along with lifestyle changes. Nevertheless, TA in these patients is often low, jeopardizing the effectiveness of such interventions. Although it is usually better than in primary prevention. In example, in a meta analysis, adherence was 50 and 66% for primary and secondary CV prevention studies, respectively (6).

In a study conducted in Spain involving 92,436 patients (62% men, mean age 72 years) with cardiovascular events, of whom 60.5% presented with stroke, 30.6% with myocardial infarction and 8.9% with revascularization were included. Drugs for secondary prevention were antiplatelets, angiotensin-converting enzyme (ACE) inhibitors/Angiotensin II receptor blockers (ARBs) and statins. Lack of adherence was defined as medication abandonment. Less than half of patients adhere to all three drugs within the first year post-event. Information was obteined from electronic medical records in real practice (7).

Multiple factors contribute to TA issues, often interrelated. Patient-related factors include age, sex, race, socioeconomic status, employment situation, forgetfulness, educational level, and personal beliefs regarding their illness, such as disagreement with the diagnosis and treatment costs. Healthcare professional-related factors encompass inadequate communication skills, insufficient follow-up, poor healthcare professional-patient relationships, and lack of clarity in prescribed instructions. Disease-related factors may involve acute or chronic conditions, comorbidities, mental health issues, and fatigue from prolonged treatments. Treatment-related factors include dosage, duration, type of recommendations (pharmacological or lifestyle changes), number of drugs, dosage forms, and side effects. Family and social environment characteristics also play a role, where supportive networks can enhance TA, while certain beliefs or cultural factors may hinder it. Lastly, healthcare system-related factors such as time constraints, frequent changes of healthcare providers, poor coordination among them, waiting lists, consultation conditions, and lack of digitalization can adversely affect TA (8).

No single intervention has been conclusively shown to improve TA independently. However, a combination of strategies may enhance adherence. Various approaches have been implemented, including health education, motivational interviewing, reminder phone calls, simplified dosing regimens, medication compliance aids (MCAs), packaging labels and pharmacological combinations such as polypills (9).

In chronic diseases, non-adherence to hygienic-dietary recommendations ranges from 70 to 95% (10), while adherence to pharmacological treatments is approximately 50% (11); however, although pharmacological treatments represent the foremost strategy currently available for the prevention and management of chronic diseases, adherence to these therapies remains below anticipated levels and continues to present significant challenges for both healthcare professionals and patients. TA also differs between patients with symptomatic and asymptomatic conditions. In asymptomatic patients, there is often a belief that they do not need the medication, which can contribute to poor adherence (12). This can vary based on age, pathology, or other factors, highlighting the magnitude of this problem. It is estimated that in secondary prevention, more than one-third of patients discontinue treatment after 6 months, and around half do so within a year (6). This is particularly relevant in CVD within primary care, where up to 39.4% of patients abandon prescribed treatments compared to 22.4% in hospital care (13).

Chronic illnesses pose substantial challenges for patients and their caregivers, particularly in the realm of disease management. The Chronic Care Model (CCM) (14) addresses these challenges by advocating for proactive, integrated, and patient-centered approaches to care and health education. It underscores the importance of patient engagement in self-management, encompassing participation in health-promoting activities, adherence to treatment protocols, and effective communication with healthcare providers. The CCM facilitates systematic assessments, preventive interventions, and psychosocial support, with the objective of enhancing health outcomes by addressing both clinical and emotional needs.

The objective of this study is to evaluate the efficacy of an intervention through health education based on the CCM implementation to increase TA in patients with secondary prevention for CVD.

## 2 Materials and methods

### 2.1 Aims and objectives

The primary aim of this study is to evaluate the efficacy of an intervention through health education based on the Chronic Care Model’s implementation to increase TA in patients with secondary prevention for CVD.

Other secondary objectives are:

- Evaluate the efficacy of an intervention based on the implementation of the CCM on the control of cardiovascular risk factors (CVRF).
- Assess whether the intervention affects the improvement of patients’ quality of life.
- Determine TA in patients undergoing secondary prevention for CVD.
- Compare differences in TA based on sex, age, race, and education level.
- Identify modifiable factors associated with poor TA.

### 2.2 Study design

Open label randomized controlled trial, selecting patients presenting the inclusion criteria through consecutive non-probability sampling. The participants will be randomly assigned to either the intervention or control groups using a random number table. An intervention will be conducted on a group of patients (intervention group), and its outcomes will be compared to those of the control group, which will receive standard follow-up under routine clinical practice conditions. The follow-up will last for 1 year, at the conclusion of which the primary and secondary variables will be assessed. Nevertheless, an evaluation will be conducted at 6 months, and if significant benefits are evident, the study will be terminated.

### 2.3 Participants

A total of 90 patients undergoing secondary prevention for CVD will be recruited from a health center in Alicante, Spain. The target population will consist of patients undergoing secondary prevention for CVD who are receiving treatment with statins, (ACE) inhibitors/ (ARBs) at a single daily dose, and acetylsalicylic acid (ASA). The inclusion criteria are: (1) adults of both sexes, aged 18 years or older, who provide written informed consent after reading the patient information sheet; (2) patients receiving daily single-dose (ACE) inhibitors/ (ARBs), ASA, and statins for at least 6 months as part of secondary prevention; (3) diagnosis of cerebrovascular disease, ischemic heart disease or peripheral vascular disease; (4) patients under regular follow-up at our health center; and (5) patients identified with suboptimal therapeutic adherence, defined by the Haynes-Sackett test supplemented with a Medication Possession Ratio (MPR) < 80% for any of the three drugs in the past 6 months. The exclusion criteria include: (1) refusal to participate; (2) incomplete consent form; (3) language barriers; (4) psychiatric, psychological, neurological, or social conditions that impair the ability to participate in the intervention or follow-up; (5) life expectancy of less than 1 year; (6) patients whose drug management is handled by a caregiver (professional or family member); (7) Barthel Index score < 60; (8) institutionalized patients; and (9) patients currently enrolled in similar research studies.

Patients from our health center will be contacted by telephone, selecting those patients recorded in the electronic medical records with a coded diagnosis according to the International Classification of Diseases, Tenth Revision (ICD-10) of cerebrovascular disease, ischemic heart disease, and peripheral vascular disease in secondary prevention for CVD, who are prescribed all three drugs and meet the inclusion criteria while not presenting any exclusion criteria outlined in the study protocol. They will be informed about the study, and those who confirm their participation will be scheduled for an in-person appointment (with a companion, if possible, to also involve them in the intervention).

During the first consultation with the principal investigator, patients will be invited to participate in the study, proceeding to verify compliance with the selection criteria. To evaluate TA, the Haynes-Sackett test will be administered, supplemented by the MPR from the medical history, reviewing the year prior to the consultation. The purpose of the research will be explained, along with informed consent and its signing. Additionally, a patient information sheet will be provided.

### 2.4 Intervention group

The intervention will consist of health education based on the recommendations of the CCM, with the aim of empowering patients for effective disease management through therapeutic education and shared decision-making. Two individual sessions will be conducted (one in the first month and another in the fourth month) and four group sessions (one each quarter, with participant numbers ranging from 5 to 10). Individual sessions will last 30 min, while group sessions will last 1 h.

In the first trimester, one individual session will be held (in the first month), followed by a group session (in the second month). In the second trimester, an additional individual session will take place (in the fourth month), along with a group session (in the sixth month), followed by subsequent group sessions scheduled for the ninth and twelfth months. These sessions will address the following components:

- Health education: emphasize the significance of TA and the objectives to be achieved, providing patients with information on the consequences of inadequate treatment adherence. Enhance patients’ understanding of their condition, potential adverse effects of drugs, how these may affect their well-being, and strategies for managing these effects. Clarify the benefits of adherence while exploring personal beliefs, concerns, and resistance to treatments, taking into account the patient’s cultural background. Highlight the importance of controlling additional CVRF, such as blood pressure, blood glucose, and weight, along with recommendations regarding the advantages of adhering to a Mediterranean diet.
- Mobile phone utilization: encourage the use of mobile phones as reminders for drug adherence through alarms and the ti.care® application.
- Drug verification: confirm that the drugs and dosages taken by the patient align with the prescribed regimen.
- MCAs: implement this system in collaboration with the patient’s community pharmacy at no cost.
- Shared responsibility: inform patients about the concept of shared responsibility and the importance of collaborative decision-making to achieve optimal health outcomes.
- Drug interchangeability: educate patients that their community pharmacist should not make substitutions between drugs bioequivalence with differing appearances.
- Empathetic approach: foster an empathetic atmosphere that avoids blame, promoting active listening and understanding.
- Empowerment: equip patients with the necessary skills to manage their own conditions, thereby fostering autonomy.

### 2.5 Control group

In the control group, patients will receive follow-up care under standard clinical practice conditions, which typically involve routine consultations with their family physician. During these visits, the emphasis is primarily on monitoring CVRF and assessing drug adherence based on the information shared by the patients. While this conventional approach provides essential support, it may not always encompass structured educational interventions or personalized strategies to increase TA.

### 2.6 Study variables

The main variable to evaluted will be TA using the Haynes-Sackett test to determine adherence vs. non-adherence (a dichotomous qualitative variable), complemented by the MPR to calculate a percentage (a continuous quantitative variable). Good TA is defined as responding that the patient experiences no difficulties taking all prescribed tablets and has an MPR > 80% for the three preventive drugs. Conversely, if these conditions are not met, it is classified as poor TA. Poor TA, based on the MPR, is indicated if the patient has not picked up 20% or more of the prescribed packages from the pharmacy in recent years. A diagnosis of poor TA can be made using either method. Since three TA are analyzed, poor TA is established if it occurs with any one of them, two, or all three.

#### 2.6.1 Secondary variables

- Group: a binary qualitative variable (intervention or control group).
- Age: a continuous quantitative variable measured in years.
- Sex: a dichotomous qualitative variable (male/female).
- Race: a nominal qualitative variable.
- Level of education: an ordinal qualitative variable.
  Illiterate.
  Elementary school studies.
  Technical studies.
  Secondary Education Studies.
  University studies.
- Degree of functional dependence in activities of daily living: a discrete quantitative variable measured using the Barthel Index.
- Quality of life: a categorical variable assessed using the EuroQol- 5 Dimension (EQ-5D). It consists of two parts: in the first, the patient evaluates their health status, and the second part utilizes a visual analog scale (VAS) from 0 to 100.

### 2.7 Statistical analysis

The data will be analyzed using version 23.0 of the Statistical Package for the Social Sciences (SPSS), developed by IBM Corp. (Armonk, NY) for Windows, and the analysis will be performed with masking of third parties.

First, a univariate descriptive analysis will be conducted for qualitative variables, expressed as percentages, and for quantitative variables, expressed as means and standard deviations, with 95% confidence intervals calculated in all cases. Additionally, a bivariate analysis will be performed to identify associations between the intervention and changes in the parameters outlined in the study objectives, with particular focus on TA. Finally, a multivariate analysis will be carried out to isolate potential confounding factors and assess the impact of the intervention on TA, adjusted for the remaining studied variables. Statistical significance will be established with a *p*-value of less than 0.05.

### 2.8 Sample size calculation

Based on a TA rate of 50% in the control group and 80% in the intervention group, with a type I error of 5% (95% confidence interval), a type II error of 20% (80% power), and accounting for a 15% loss to follow-up, the final sample size will be of 45 patients per group.

## 3 Discussion

To the best of our knowledge, this study represents one of the few randomized controlled trials implementing an intervention similar to the one proposed in this research, based on CCM, specifically tailored to this context and population.

The trial aims to compare an intervention to increase TA in patients with secondary prevention for CVD with standard follow-up under routine clinical practice conditions.

The lack of TA in chronic diseases, particularly in CVD, is a global issue due to the increasing prevalence of comorbidities and decreasing life expectancy. Moreover, if patients do not adhere adequately to their treatment regimens, physicians may implement unnecessary therapeutic modifications, such as increasing dosages or prescribing more potent or expensive medications, and may even request unnecessary additional tests. Consequently, the WHO identifies this as the primary cause for the failure to achieve the expected benefits from treatments, ultimately resulting in diminished quality of life and increased healthcare costs.

In acute pathology, it is observed that TA tends to be higher because patients transition from a healthy state to an ill one, typically accompanied by significant symptoms. Consequently, they become much more aware of the limitations and the short-term impact that the illness has on their quality of life. However, in chronic diseases, the perceived gains in health diminish, particularly beyond 6 months post-cardiovascular event, as patients adapt to their new circumstances and find it challenging to discern the benefits of treatments, which are often preventive and do not directly alleviate symptoms or signs (15–17).

Regarding the applicability and utility of the predictable outcomes from our study, we believe that due to the low TA observed in patients with CVD, the relevance of these results would be significant. We propose a feasible intervention within primary care to address a public health issue that has considerable implications, such as increased morbidity and mortality, decreased quality of life, and substantial healthcare costs, among others.

Therefore, should our hypothesis be corroborated, it would be pertinent to evaluate the implementation of this intervention aimed at improving TA in patients undergoing secondary prevention for CVD, particularly concerning the three preventive drugs. Additionally, patients undergoing secondary cardiovascular prevention are at high risk of new cardiovascular events, often with a worse prognosis than the initial one. Modifying CVRF can lead to a significant reduction in both overall cardiovascular risk and mortality in these patients. As a result, this intervention not only seeks to improve TA but also to achieve better control of CVRF, potentially resulting in a positive impact on clinical outcomes and a reduction in future adverse events.

This study has several limitations and biases. To minimize random error, accounting for a 15% loss to follow-up, which was accounted for in the sample size calculation to address potential attrition during the study. Regarding information bias, TA was assessed using the Haynes-Sackett test and MPR. Utilizing a single method could lead to an overestimation of TA; therefore, the MPR was employed as an additional method to minimize this risk. Nevertheless, there is no optimal method for evaluating TA.

Moreover, as this is a research study, it may influence patients’ attitudes due to attention bias or the Hawthorne effect, potentially altering their behavior during participation (for instance, by retrieving drug from the pharmacy but not actually taking it) or improving TA simply because they feel observed. To address this, a control group is included, where the effect may also be present. In any case, as is common in studies, an increased TA may occur compared to the usual rate, but it would not impact the effect of the intervention if a benefit is demonstrated.

Additionally, this is a single-center study, which may limit the extrapolation of results to other healthcare settings or countries with different health systems; however, it allows for better control of the intervention and measurements. If the results are favorable, multicenter studies will be proposed to facilitate the generalization of the conclusions. In any case, the study is conducted under standard clinical practice conditions, which aids in extending the findings to other centers.

Furthermore, this is an open-label clinical trial, as the intervention cannot be masked. Nevertheless, efforts will be made to ensure that the care received by patients in both groups is equivalent since it takes place in standard clinical practice conditions. Open-label studies have this limitation, which is generalizable to all of them.

Selection bias may occur due to factors such as sex, age, race, and educational level. To minimize this bias, random assignment to intervention and control group will be implemented.

Finally, the high workload of Family and Community Medicine Specialists may pose a limitation for patient recruitment. However, the study will involve motivated professionals who specialize in cardiovascular pathology, and the majority of the activities will be conducted outside regular consultation hours.

## 4 Ethics aspects

The study will be conducted in accordance with Spanish legislation, the Declaration of Helsinki (WMA, 2008), and the Good Clinical Practice Guidelines, along with Regulation 2016/679 of the European Parliament and of the Council of April 27, 2016, concerning the processing of personal data, and any applicable norms or legislation.

Data will be collected in a coded manner within the Case Report Form (CRF) designed for the study, and this CRF will be cross-referenced with identifying data, which will be safeguarded by the principal investigator. The CRF only will be accessible to the principal investigator and the statistician.

In accordance with current legislation regarding research, the completion of the written informed consent form will be necessary before participating in the study, along with the provision of a patient information sheet.

Furthermore, this study protocol has a favorable dictamen to the Institutional Review Board of Dr. Balmis General University Hospital of Alicante, Spain (approval date: 12 February 2024; code: 2024–016) and code of responsible research from the Miguel Hernández University of Elche (ADH.SPU.DLOB.FMLM.24). The study was registered at clinicalTrials.gov with the identifier of NCT06510946 on 19 July 2024.
